# Risk factors associated with asbestos-related diseases: a community-based case–control study

**DOI:** 10.1186/1471-2458-13-723

**Published:** 2013-08-06

**Authors:** Magdalena-Isabel Rosell-Murphy, Rafael Abós-Herràndiz, Josep Tarrés Olivella, Constança Alberti-Casas, Isabel García Allas, Xavier Martinez Artés, Ilona Krier Günther, Isidre Grimau Malet, Ramon Orriols Martínez, Jaume Canela-Soler

**Affiliations:** 1 Primary Care Team Serraparera, Catalan Institute of Health, Cerdanyola del Vallès, Spain; 2 Primary Care Team Pinetons, Catalan Institute of Health, Ripollet, Spain; 3 Primary Care Team Canaletes, Catalan Institute of Health, Cerdanyola del Vallès, Spain; 4 ICAM, Department of Health Evaluation, Barcelona, Spain; 5 Primary Care Team Rosa dels Vents, Catalan Institute of Health, Barberà del Vallès, Spain; 6 Palliative Care Unit, Consorci Hospitalari Parc Taulí, Sabadell, Spain; 7 Respiratory Medicine Department, University Hospital Vall d'Hebron - CIBER Respiratory Diseases (CIBERES), Catalan Institute of Health, Barcelona, Spain; 8 Public Health Department, Universitat de Barcelona, Barcelona, Spain; 9 Department of Epidemiology and Biostatistics, College of Public Health, University of South Florida, Tampa, USA

**Keywords:** Asbestos-related diseases, Primary health care, Mesothelioma, Case–control study

## Abstract

**Background:**

Asbestos is a first level carcinogen. However, few epidemiological studies analyse the risk and protective factors associated with asbestos-related diseases and follow up these conditions in the general population. Pleural mesothelioma, caused by inhalation of asbestos fibres at work, at home or in the environment, is the most representative asbestos-related disease.

The objectives of this study are to analyse the risk and protective factors associated with asbestos-related diseases and to investigate the incidence of new clinical manifestations in patients already diagnosed with some form of ARD.

**Methods/Design:**

We have designed a matched case–control study with follow up of both cohorts from a population of a health district of the Barcelona province that has been exposed to asbestos for a period of 90 years.

**Discussion:**

A better understanding of asbestos-related diseases should improve i) the clinical and epidemiological follow up of patients with this condition; ii) the design of new treatment strategies; iii) and the development of preventive activities. At the end of the study, the two cohorts created in this study (affected cases and healthy controls) will constitute the basis for future research.

## Background

Inhalation of asbestos fibres is detrimental to human health. The toxicity of asbestos has been known since the early twentieth century and linked almost exclusively to pleural mesothelioma [1]. However, this is just one of the 10 clinical conditions that currently define Asbestos Related Diseases (ARD) [2].

Asbestos is a mineral whose main characteristics are incombustibility and thermal isolation. As a result of these properties, asbestos is used in a wide range of applications in almost every industrial sector [3,4].

Workers at fibre cement plants, painters, carriers of asbestos materials and construction workers are the population with the highest risk of exposure to asbestos fibres [5].

ARD is a group of 10 conditions that originate from the inhalation and subsequent deposit of asbestos fibres in the pulmonary parenchyma and which affect mainly the respiratory system. Following the most widely accepted guidelines [2,6], ARD are classified in two groups (malignant and non-malignant) which include diseases of the lung parenchyma, the pleura and the bronchi.

Asbestos toxicity is related to its fibrous structure, since studies have demonstrated that pulverized asbestos does not cause disease [7]. Due to individual susceptibility factors, the development of ARD cannot be ruled out in individuals with low intensity exposure to asbestos [8,9]. The study of risk and protective factors of ARD can be hindered by the latency period, which can be as prolonged as 40 years [10].

In addition to its fibrogenic properties, asbestos is a carcinogen. [4] The most widely accepted oncological model is the dose–response without a safety level. [8,11] Fibrogenic and carcinogenic properties are related, particularly in the amphibole type of asbestos. The role of amphibole asbestos as a co-carcinogenic agent has been shown in the development of mesothelioma and in other pulmonary neoplasms [12]. The risk of malignant mesothelioma increases with age and depends on when first exposure started. These factors are also closely associated with the level of exposure, continuity of exposure, and latency period [13].

The main risk factor to develop ARD is inhalation of asbestos fibres as a result of work exposure. However, not everybody that has been or is exposed to asbestos develops ARD. A main difficulty in the study of ARD is the lack of information related to exposure history in the medical records and the lack of awareness of the workers themselves. In addition, the late age at diagnosis due to the time lag between exposure and clinical manifestations is another factor that explains the significant number of ARD cases that are not considered a work-related disease [14]. A large number of ARD worldwide are still not considered or notified as a work-related disease [6].

Asbestosis, asbestos-related lung cancer and mesothelioma are classified in Spain as work-related diseases in the Royal Decree 1995/1978 of 12th of May [15]. Nevertheless, the actual morbidity and mortality caused by ARD are still largely unknown, mainly due to the scarce notification of these cases as work-related conditions [16].

Fibre inhalation when living near an asbestos source, living together with an asbestos worker that brought asbestos fibres in the work clothes, the domestic use of products manufactured with asbestos and simple environmental exposure constitute non-work related exposures that can also cause ARD. The association with simple environmental exposure is better known for mesothelioma than for asbestos-related lung cancer [17-20]. The incidence of benign ARD cases that originate from environmental exposure has hardly been investigated. Moreover, no data on the incidence of additional ARD in patients already suffering from any form of ARD exist, and no studies on the protective factors of ARD have been published.

The association of asbestos with ARD is modified by tobacco consumption. Indeed, smokers exposed to asbestos are at higher risk of developing radiologic signs of asbestosis than non-smokers [21]. Also, asbestosis itself is a risk factor for lung cancer [12]. To date, no definite association between tobacco consumption and mesothelioma has been found [4,9].

It is generally accepted that the spontaneous incidence of mesothelioma is very rare, close to 1 new case/million inhabitants/year [22]. However, this figure has been now reviewed and increased to 3 new cases/million inhabitants/year [7]. In the province of Barcelona, the incidence of mesothelioma estimated from mortality data of residents equals 8.3 cases/million inhabitants/year in men, and 4.7 cases/million inhabitants/year in women. These figures are much higher in Barcelona’s neighbouring towns of Cerdanyola del Vallès, with 18.4 cases/million inhabitants/year, and Ripollet, with 17.6 cases/million inhabitants/year [23].

A recent study on all types of ARD in our area show an incidence of 95 cases/million inhabitants/year and a prevalence of 910 cases/million inhabitants/year; in particular, the incidence and prevalence of mesothelioma are 30 and 10 cases/million inhabitants/year, respectively [10]. The severity of pleural mesothelioma and the possibility of an upward trend in the incidence of ARD [24] are the leading motives for the research team to undertake the current project, in continuity with our previous work.

The plausibility of asbestos as the cause of ARD will be strengthened with the case–control design of this project. Also, the creation of two parallel cohorts for comparison over time will constitute the basis for future research.

Asbestos-related diseases, and non-malignant ARD specifically, are poorly understood causes of morbidity and mortality. Indeed, the course of these conditions in our study population is unknown. The transition of ARD from non-malignant to malignant has not been elucidated, and few prospective studies that address this question have been published [25].

Up-to-date data on the impact of asbestos exposure on morbidity and mortality and on the risk and protective factors of the exposed general population are needed. Our working hypothesis is that within the group of ARD patients that develop a new ARD, a significant number of non-malignant ARD cases will develop into malignant ARD. The pattern of geographical distribution of new ARD cases requires a study with a case–control design.

The experience of the research team suggests that over 75% of ARD cases are diagnosed within the primary care network, since it is the best and most accessible gateway to the National Health System. Thus, the primary care network becomes a comprehensive setting to conduct this type of study.

The analysis of mortality in ARD cases is another relevant aim of this project. In addition to the difficulties in obtaining a final certain diagnosis, ARD must compete with other diseases as the underlying cause of death certification.

The concordance between the underlying cause of death in the Statistical Bulletin and final hospital discharge records is a fundamental quality measurement of our National Health System [26]. Health services use within the National Health System is an essential research area. However, in our setting it is as yet underdeveloped for primary care [27], particularly in relation to ARD. We therefore aim to analyze the impact of ARD on health services through direct costs (financial burden for high cost health conditions) using the prevalence method, together with burden of disease, which combines mortality with health outcomes [28].

### Hypotheses

The study of risk and protective factors associated with ARD, new ARD in patients with one or more previous diagnosis of ARD (additional ARD) and the space-time clustering of ARD patients in specific geographical areas generate the following working hypotheses:

1. Lifestyles and individual susceptibility influence the course of asbestos-related diseases.

2. Clinical parameters will change in 50% ARD patients every year, and for the three years of duration of the study.

3. 10% of patients diagnosed with one or more ARD will present some new ARD (additional ARD) during the three years of the study.

4. Environmental exposure determines the geographical clusters of ARD cases in the study area.

5. The protocolized follow up of all ARD patients facilitates the management of the disease and decreases the health expense allocated to ARD (burden and costs of the disease).

6. ARD as the underlying cause of death certification coincides with the mortality found in the population of the study area.

### Objectives

#### Main objective

The main objective of this project is the identification of protective and risk factors associated with new cases of ARD and with additional ARD, and to characterise ARD’s geographical clustering.

#### Specific objectives

– To identify the lifestyles and associated morbidity that might determine ARD incidence.

– To quantify the proportion of ARD cases which present changes in the clinical parameters on a yearly basis during the three years of duration of the study.

– To quantify the proportion of ARD cases that present an additional ARD during the three years of duration of the study.

– To describe the geographical clustering of ARD cases in the study area, determined by exposure factors and individual susceptibility.

– To analyze the effectiveness of the management and control of ARD through a specific protocol and its impact in the reduction of the health budget allocated to ARD (burden and costs of ARD).

– Finally, to describe the level of agreement between ARD as the underlying cause of death stated in death certificates and final hospital discharge records.

## Methods/Design

1. **Study area:** health district with the 5 towns in the Barcelona province with the highest morbidity and mortality caused by asbestos [10]: Cerdanyola del Vallès, Ripollet, Barberà del Vallès, Badia del Vallès and Montcada i Reixac. All these towns refer the ARD patients to the only hospital that participates in this project (Hospital Parc Taulí).

2. **Study population:** all inhabitants (approximately 174,515 [29]) of the study area that consent to participate in the study.

3. **Study network setting:** all Catalan Health Institute Primary Health Care Teams (PHCT) in the 5 above mentioned towns. They all use the same classification of disease (ICD-10) and the same referral hospital. In total, this health services network provides health care for 174,515 people.

4. **Design:** community study of ARD incident cases and matched controls (1:1), with yearly, protocolized follow up of ARD cases and healthy controls.

5. **ARD definition:** ARD is a group of 10 conditions that affect mainly the respiratory system, caused by the inhalation and subsequent deposit of asbestos fibres. Following the most widely accepted guidelines [2], ARD are classified into malignant (pleural mesothelioma, peritoneal mesothelioma, asbestos-related bronchopulmonary carcinoma, and other rare neoplasms) and non-malignant, which comprise diseases of the lung parenchyma (asbestosis or interstitial lung fibrosis), of the pleura (isolated pleural plaques, diffuse pleural fibrosis or pleural thickening and benign pleural effusion), and of the bronchi (chronic bronchial obstruction and rounded atelectasis).

6. **Definition of ARD case:** cases are defined as any person within the study area diagnosed with at least one ARD between 1/1/2011 and 31/12/2013, in agreement with the diagnostic criteria internationally accepted [2]. All participants must sign the informed consent form. For the diagnosis of ARD, the fulfilling of the three following conditions is considered necessary and sufficient: a) imaging or pathology techniques such as the presence of asbestos bodies in the bronchoalveolar lavage or in a cytology sample must show that the patient has a lesion compatible with ARD in the respiratory system; b) sufficient time elapsed between work and/or environmental asbestos exposure; c) and exclusion of other possible causes.

7. **Definition of control:** when a patient is diagnosed with ARD, he/she will be assigned a matched control according to the following criteria: no ARD diagnosis, same age (±5 years), same gender, same town with same period of residence (exposure), and accepts the ethical requirements of the study. The controls that become a case during the study period will be followed up and analysed in depth.

8. **Study variables:**

Essential variables:

a) ARD diagnosis (10 conditions): interstitial lung fibrosis, isolated pleural plaques, diffuse pleural fibrosis, benign pleural effusion, chronic bronchial obstruction, rounded atelectasis, pleural mesothelioma, peritoneal mesothelioma, asbestos-related pulmonary carcinoma and other neoplasms.

b) Clinical course: stable, worsening, detection of new ARD, change in disease progression phase, death.

Socio-demographic variables: gender (female = 1, male = 0), age, place of birth and current place of residence.

Exposure variables: source (asbestos factory, other related businesses, living near an asbestos source, living with an asbestos worker and unknown) and exposure times.

Clinical variables: tobacco consumption (non smoker/smoker/ex-smoker); cigarettes/year; pack-years of smoking; years since quitting for ex- smokers; comorbidity, i.e., polymorbidity and current medication classified by organic systems – cardiovascular, respiratory, digestive, nephro-urologic, dermatological, musculoskeletal, nervous and oncologic; and variables related to physical activity in adults [30,31].

ARD follow up variables:

a) Clinical symptoms: asymptomatic; persistent dry cough; respiratory failure symptoms; oncologic symptoms; other.

a) Clinical signs: digital clubbing, crackles.

a) Imaging signs: pleural plaques; thickening of the pleura; rounded atelectasis; pulmonary fibrosis; partial pleural effusion; massive pleural effusion.

Diagnostic variables: imaging (X-ray, conventional and high-resolution computed axial tomography, magnetic resonance imaging, ultrasound); pathology (biopsy, cytology, autopsy); respiratory function tests (spirometry, gas exchange).

Variables of health resources utilization: visits to the family doctors and specialists and scheduled and unscheduled hospital admissions, per year and due to any disease.

9. **Circuit for cases and follow up:** each Primary Health Care Team (PHCT) has a study assigned physician with an interest in ARD that will be responsible for the protocolized follow up of each case. There will be a first visit in the practice followed by two contacts, usually via telephone interview. The family doctor that diagnoses the patient refers him/her to the respiratory medicine specialist for confirmation, then includes all this information in the electronic medical record and notifies the assigned physician, who will then interview the patient and if he/she is eligible, the study assigned physician will explain the study and invite him/her to participate. If the patient accepts, he/she must sign the informed consent form and will be included in the study.

10. **Circuit for controls and follow up:** controls will be selected randomly, based on each case’s characteristics and with the assistance of a health information system. If the control selected cannot be reached or refuses to participate, the next person in the list according to a table of random numbers will be contacted.

11. **Level of detection of cases:** a major challenge of this study is the comprehensive detection of incident cases, which will be obtained through the optimisation of the circuits described in sections 9 and 10. To this end, the ability of the research team to train and raise awareness among the colleagues in primary care in ARD detection is considered essential. A minimum of one session per year will take place for the three years of duration of the project, to train and involve the professionals of the primary care teams of the study area. Videoconferences can also be used. The research team will identify new cases from the hospital register.

12. **Estimated number of new cases detected and of controls:** according to previous data from the current study area [10], 17 new cases (51 during the three year study period) will be detected annually amongst this population of 174,515 inhabitants.

13. **Collected data entry in a single database:** data will be automatically entered using a bar code reader. A hard-copy of every file will be stored for future checks, together with the informed consent form.

14. **Statistical analysis:** a descriptive analysis of the variables will be carried out. The differences between groups will be analysed with the chi-square test, Student’s t-test, ANOVA or the corresponding non-parametric tests. The crude association between dependent and independent variables will be analysed using the Mantel-Haenszel’s matched odds ratio. Conditional logistic regression will be used to calculate the adjusted odds ratio. For the survival analysis of the follow up of the cohort Cox regression will be used, and the hazard ratios will be calculated. Mortality underreporting will be calculated through the analysis of the death bulletins for the patients affected by ARD who die as a result of any of the 10 ARD conditions during follow up. Principal Component Analysis will be used for the analysis of geographical clusters. For the economic evaluation, regression methods will be used taking into account direct costs (health resources utilized), indirect costs (production lost) and burden of disease (mortality and objective and perceived health status). The significance level will be set at 5%. Stata v11 and SPSS v21 will be used for the analysis.

15. **Ethics:** cases and controls will be requested to sign the informed consent form. They will be explained the general and specific ethical considerations of the study with regard to the right to privacy, anonymity, confidentiality, cancellation and information in accordance with the principles of the Declaration of Helsinki. The study has been approved by the Clinical Research Ethics Committee of the Primary Health Care University Research Institute (IDIAP) Jordi Gol.

## Discussion

The main limitations of this study are the memory bias of patients and controls and/or their relatives, the time elapsed between initial exposure to asbestos and the onset of ARD, and the different degrees of quality in the information obtained. Precision in recording the variables that refer to time, place and lifestyles is the best tool to minimise these limitations. Also, the health information system that encompasses the entire population of the study area is a powerful, effective tool to easily identify possible cases and controls that will be made available to the investigators.

ARD is an underreported, notifiable disease, and it is rarely found as the underlying cause in death certificates. In addition, it is an underreported work-related condition.

Other limitations are the refusal of the population to participate in this type of study. Adequate training of the professionals who participate in the study will circumvent most aforementioned limitations.

The case–control design of this study presents a major advantage to investigate conditions with a low incidence. The comparison of outcomes between groups underscores the health impact of continuous asbestos exposure in the community.

## Conclusions

This study will describe the role of less known factors involved in the protection and risk of ARD development and progression. The study of comorbidities and lifestyle of these patients, variables routinely collected in the information systems of primary care, is of particular interest.

The follow up of these patients in their own primary care practices will enable the detection of ARD in previously unaffected patients and also of disease progression in those patients previously diagnosed with some form of ARD by means of modifications in their clinical parameters.

Information on the general characteristics of patients suffering from ARD and on the geographical clustering of patients that originates from an intense and well known source of exposure will improve our understanding of the impact caused by asbestos on human health. The set up of two cohorts for a case–control study will facilitate time and survival comparisons. With the publication of this protocol, public health systems will have access to a more precise tool in the fight against asbestosis.

## Abbreviations

ARD: Asbestos related diseases; PHCT: Primary health care team.

## Competing interests

The authors declare that they have no competing interests.

## Authors’ contributions

MRM and RAH are responsible for the study design and writing the protocol; RAH, CAC, JCS and MRM reviewed the literature and contributed to the manuscript’s introduction, definition of variables, methodology and analysis; XMA and CAC are responsible for the design and maintenance of the patients’ database; JTO, ROM, IGA and IGM contributed to the supervision and final draft of the manuscript. All authors read and approved the final manuscript.

## Pre-publication history

The pre-publication history for this paper can be accessed here:

http://www.biomedcentral.com/1471-2458/13/723/prepub
